# Atrazine-induced apoptosis of splenocytes in BALB/C mice

**DOI:** 10.1186/1741-7015-9-117

**Published:** 2011-10-27

**Authors:** Xiaofeng Zhang, Mingqiu Wang, Shuying Gao, Rui Ren, Jing Zheng, Yang Zhang

**Affiliations:** 1 Department of Toxicology, College of Public Health, Harbin Medical University, Harbin, Heilongjiang Province, 150081, PR China; 2 Institute of Toxicology, Centre of Disease Control of Heilongjiang Province, Harbin, Heilongjiang Province, 150030, PR China; 3 Institute of Monitoring for Public Health, Centre of Disease Control of Heilongjiang Province, Harbin, Heilongjiang Province, 150030, PR China

## Abstract

**Background:**

Atrazine (2-chloro-4-ethytlamino-6-isopropylamine-1,3,5-triazine; ATR), is the most commonly applied broad-spectrum herbicide in the world. Unintentional overspray of ATR poses an immune function health hazard. The biomolecular mechanisms responsible for ATR-induced immunotoxicity, however, are little understood. This study presents on our investigation into the apoptosis of splenocytes in mice exposed to ATR as we explore possible immunotoxic mechanisms.

**Methods:**

Oral doses of ATR were administered to BALB/C mice for 21 days. The histopathology, lymphocyte apoptosis and the expression of apoptosis-related proteins from the Fas/Fas ligand (FasL) apoptotic pathway were examined from spleen samples.

**Results:**

Mice administered ATR exhibited a significant decrease in spleen and thymus weight. Electron microscope histology of ultrathin sections of spleen revealed degenerative micromorphology indicative of apoptosis of splenocytes. Flow cytometry revealed that the percentage of apoptotic lymphocytes increased in a dose-dependent manner after ATR treatment. Western blots identified increased expression of Fas, FasL and active caspase-3 proteins in the treatment groups.

**Conclusions:**

ATR is capable of inducing splenocytic apoptosis mediated by the Fas/FasL pathway in mice, which could be the potential mechanism underlying the immunotoxicity of ATR.

## Background

Atrazine (2-chloro-4-ethytlamino-6-isopropylamine-1,3,5-triazine; ATR) is the most extensively used broad-spectrum herbicide in the world. Scientists from many countries are increasingly concerned and interested in ATR due to its adverse environmental effects. Residues of ATR, its primary metabolite, deethylatrazine, and other derivatives of the parent compound can leach from soils and persist in ground and surface water for several years [1,2]. It is frequently detected as an environmental pollutant at concentrations exceeding the maximum containment level as set by the US Environmental Protection Agency (EPA) [3]. Occupational exposure of farmers and other agricultural workers to high concentrations of ATR is of particular concern. Levels of ATR exposure can be detected in the saliva and urine of these workers after spraying. It is not only the direct ATR applicators at risk, but also their families through detectable levels in body fluids. Considerably higher amounts of ATR and its metabolites are found in the urine in populations living within proximity of farms that use this herbicide [4]. ATR has also been found in the urine of children of non-agricultural families, demonstrating non-occupational exposures [5].

Many toxicological studies of ATR focus primarily on the effects of ATR on the endocrine and reproductive systems. ATR affects levels of steroid hormones and interferes with the critical pathways for sex-specific physiological and behavioral development. This includes activation of the hypothalamic-pituitary-adrenal axis and disruption of related androgen-mediated processes, which results in demasculinization and feminization of male amphibians and rodents [6-9]. Prenatal and lactational ATR exposure affects the health and development of the male offspring in rats [10-12]. Further studies show that ATR increases aromatase activity by binding to and inhibiting phosphodiesterase, which elevates cAMP production and ultimately increases estrogen production [13-15]. Exposure to ATR results in dopaminergic neurotoxicity by disrupting vesicular storage and/or cellular uptake of striatal dopamine (DA), as well as targeting the nigrostriatal dopaminergic pathway, which decreases intracellular DA concentrations [16-20]. There is growing evidence suggesting that ATR can induce oxidative stress and DNA damage [21-23].

While there are numerous studies regarding the detrimental effects of ATR on endocrine and reproductive development, studies assessing the immunotoxic potential of ATR are scarce. A single oral dose of ATR causes transient suppression of IgM production and T cell proliferation in adult mice [24]. Brodkin *et al*. [25] suggested that exposure to ATR affects the innate immune response in frogs. Oral administration of ATR for 14 days is immunotoxic in mice, manifesting as an increase in the number of CD8^+ ^T cells and an increased cytotoxicity of T cells. A decrease of thymus and spleen weights, and total spleen cell numbers are also observed in C57BL/6 mice after daily oral treatment for 14 days at up to 250 mg/kg/day or 500 mg/kg/day [26,27]. Studies on the immunotoxicity effects of ATR have mainly been focused on the evaluation of immunity function. Less attention has been directed toward the mechanisms by which these functions are modulated.

The active apoptotic pathway in the immune system is regulated by Fas/Fas ligand (FasL) cytokine death receptors in lymphoid cells [28,29]. The Fas (also known as: CD95 or Apo-1) receptor protein crosslinks with its ligand FasL (or CD95L) and leads to the assembly of a death-inducing signal complex initiating caspase-mediated apoptosis [30,28]. The Fas receptor and ligand system is widely distributed in active B and T lymphocytes. Binding of FasL to Fas-bearing cells serves important immune system functions through activation-induced cell death; this system is also an effector of cytotoxic T cell and natural killer cell activities. Following infection, many B or T lymphocytes can become useless or harmful after their receptor genes have been rearranged. Homeostatic immunity is regulated by FasL mediated apoptosis eliminating these antigen-specific lymphocytes in excess, which regulates a steady-state supply of lymphocytes in periphery tissues. Immunotoxicity leading to the negative modulation via overexpression of Fas regulatory genes, however, can cause excess apoptosis of lymphocytes, which suppresses cellular and humoral immune responses [31,32].

Liu *et al*. [33] demonstrated ATR induced apoptosis in fish cells. Singh *et al*. [21] identified an ATR induced genotoxic effect, but no study has yet to report on ATR induced apoptosis in mammals. This study explores the effects of ATR-induced apoptosis in splenic lymphocytes on Fas/FasL proteins involved in the apoptotic pathway. This study provides evidence of a possible mechanism by which ATR-induced immunotoxicity can occur in a rodent or, more generally, a mammalian model.

## Methods

### Chemicals and reagents

Atrazine (CAS Registry Number: 301-49A; 2-chloro-4-ethylamino-6- isopropylamino-s-triazine, ATR, 98% purity) was obtained from ChemServices (West Chester, PA, USA). β Actin rabbit polyclonal antibodies, Fas, FasL and caspase-3 rabbit monoclonal antibodies, as well as the alkaline phosphatase-conjugated secondary antibodies were acquired from Santa Cruz Biotechnology (SantaCruz, CA, USA). The annexin-V/propidium iodide (PI) Kit was purchased from Pharmingen (Becton Dickinson Company, NJ, USA). ATR solutions (5 mg/ml, 10 mg/ml and 20 mg/ml) were prepared by dissolving ATR in 3% starch solution. All the solutions were kept at 4°C for a maximum of 1 week.

### Animals and treatment

Male and female BALB/C mice (16-18 g) were purchased from Tumor Hospital of Heilongjiang Province (Harbin, China). The animals were treated in accordance to the criteria outlined in the Guide for the Care and Use of Laboratory Animals prepared by National Institutes of Health. After acclimatization for 1 week, the mice were housed in standard polyethylene cages, in groups of five mice per cage and per sex, with wood shavings as bedding and given purified water and rodent chow *ad libitum*. Animal rooms were maintained at a 12 h light/dark cycle at 22 ± 2°C and 50% ± 15% relative humidity. Male and female mice were randomly divided into four groups by body weight (five mice/sex/group). Animals were treated by a daily gavage of 20 μl/g body weight at doses of 0 (3% starch solution, vehicle control) or 100, 200 and 400 mg/kg ATR for 21 consecutive days.

### Body weight and organ weight

Body weight was recorded once a week. Upon being killed after 21 treatment days, the spleen, thymus, liver and kidney were removed aseptically and weighed. The relative weight of the organs of each mouse was calculated as organ weight (mg)/body weight (g).

### Preparation of splenocytes

Splenocytes were harvested as described in Gao *et al*. [34]. The spleen was removed at the time of killing and a single-cell suspension was prepared by forcing spleen through a 400 μm stainless steel mesh. Erythrocytes were lysed with hypotonic buffered solution and lymphocytes were washed with Hanks' balanced salt solution (HBSS) prior to being resuspended in RPMI 1640 medium. Viable cells were counted by the trypan blue exclusion method using a hemocytometer. Finally, cells were adjusted into different concentrations with RPMI 1640 medium supplemented with 10% fetal calf serum.

### Histopathological examination

#### Light microscope

After fixation with 10% buffered formalin, the spleen was dehydrated, processed, and embedded in paraffin. Serial sections (5 μm) were prepared and stained with Harris's hematoxylin and eosin (H&E) for histopathological evaluation by light microscope.

#### Transmission electron microscope

The spleen was cut into 1 mm^3 ^size cubes and fixed in 1% freshly made paraformaldehyde with 2% glutaraldehyde for 24 h. Samples were fixed for 2 h in 1% osmium tetroxide and dehydrated in graded ethanol and embedded in araldite. Ultrathin sections were cut and stained with uranyl acetate and lead citrate, and then observed with transmission electron microscope (JEM-101, Jeol Electron Inc., Tokyo, Japan).

#### Apoptosis assay

Apoptosis was assessed by using annexin-V/PI Kit following manufacturer's instructions as described in Gao *et al*. [34]. Briefly, spleen lymphocytes were collected as above, adjusted to 2×10^6 ^cells/ml, then treated with a final concentration of 80 mg/ml RNase I, and stained with 50 mg/ml PI and 50 mg/ml annexin-V/fluorescein isothiocyanate (FITC) for 15 min at room temperature in the dark and analyzed by Flow Cytometry (Becton Dickinson, Franklin Lakes, NJ, USA) equipped with Cell-Quest software analysis. A minimum of three independent experiments were performed in each assay.

#### Western blot

Western blot analyses for the expression of Fas, FasL, active caspase-3 and β actin were performed as described in Zhang *et al*. [35]. Spleen was homogenized in ice-cold 50 mM Tris-HCl buffer (pH 7.4) containing 0.25 M sucrose and a 1% protease inhibitor cocktail. The homogenate was centrifuged at 4°C, 10,000 *g*, for 10 min and the supernatant was collected. Protein concentrations were determined by the Bradford protein assay. Equal amounts of protein were resolved by 10% (v/v) sodium dodecyl sulfate polyacrylamide gel electrophoresis (SDS-PAGE), followed by electrophoretic transfer to Immobilon-P membranes. Blots were blocked in Tween 20 Tris-buffered saline containing 3% (w/v) non-fat dry milk for 1 h at room temperature. Membranes were incubated over night at 4°C with an appropriately diluted primary antibody and were then incubated with an appropriately diluted secondary antibody in blotting buffer for 1 h with agitation at room temperature. Visualization of the proteins was performed by an enhanced chemiluminescence system (Amersham Biosciences, Piscataway, NJ, USA) according to manufacturer's protocol. Density of bands was quantified by densitometry. β Actin was used to normalize the samples.

### Statistics

Data were analyzed using SPSS (Chicago, IL, USA) by one-way analysis of variance (ANOVA) followed by Dunnett's multiple comparisons testing to identify significant differences between groups. All data were expressed as mean ± SD. Differences were regarded as significant at the value of *P *<0.05.

## Results

### General state of mice

All animals survived until the end of the study. There were no overt changes in appearance and/or behavior, as well as no significant differences in body weight (data no shown) in the male and female mice following ATR exposure.

### Toxic effect of ATR on the spleen

Mean spleen and thymus weight and their relative weight decreased in the mice of both sexes in the 200 and 400 mg/kg ATR treatment groups (*P *<0.05), compared to control group, as shown in Table 1. There were no significant alteration of weight and relative weight of liver and kidneys (data no shown).

**Table 1:** Summary weights of spleen and thymus in mice

Group	Spleen weight (mg)	Spleen relative weight (mg/g)	Thymus weight (mg)	Thymus relative weight (mg/g)
Control group	92.2 ± 2.50	4.02 ± 0.407	46.6 ± 2.46	2.05 ± 0.220

100 mg/kg ATR	92.0 ± 1.92	3.99 ± 0.521	44.7 ± 1.25	1.95 ± 0.395

200 mg/kg ATR	79.4 ± 1.74*	3.44 ± 0.312*	41.7 ± 0.62*	1.83 ± 0.450*

400 mg/kg ATR	71.4 ± 2.01*	3.16 ± 0.372*	40.4 ± 1.32*	1.78 ± 0.254*

Each value represents mean ± SD (n = 10, males and females combined).

*P <0.05 compared to control group.

ATR = atrazine (2-chloro-4-ethytlamino-6-isopropylamine-1,3,5-triazine).

**Figure 1:**
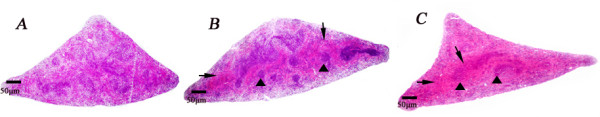
**Histopathological cross sections showing light microscopy of spleen in mice treated with atrazine (2-chloro-4-ethytlamino-6-isopropylamine-1,3,5-triazine; ATR) (hematoxylin and eosin (H&E) stain, magnification ×10)**. **(a) **Control group. The histological structure is normal. **(b) **The 200 mg/kg ATR group. Atrophic and effaced splenic germinal centers, the diminution of white pulp (filled triangles) and congestion of red pulp (arrow) in the spleen can be seen. **(c) **The 400 mg/kg ATR group. Effaced splenic germinal centers and the diminution of white pulp (filled triangles), associated with obvious congestion in red pulp (arrow).

**Figure 2:**
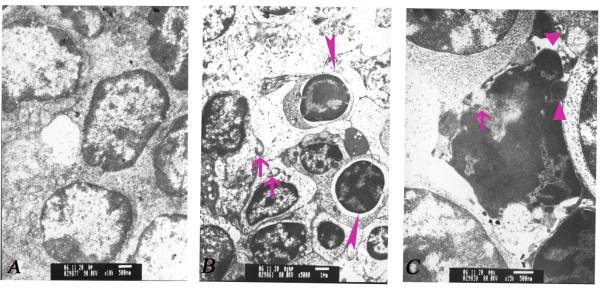
**Transmission electron microscope images showing the ultrastructural properties of splenocytes from mice treated with atrazine (2-chloro-4-ethytlamino-6-isopropylamine-1,3,5-triazine; ATR)**. **(a) **Control group. Splenocytes showing normal phenotype (magnification ×10,000). **(b) **The 200 mg/kg ATR group. Splenocytes exhibiting chromatin margination and condensation into dense granules or blocks (filled triangles), as well as mitochondrial vacuolization (arrow) (magnification ×5,000). **(c) **The 400 mg/kg ATR group. Splenocytes exhibiting apoptotic morphology including chromatin margination, mitochondrial vacuolization (arrow) and formation of apoptotic bodies (filled triangles) (magnification ×15,000).

The histopathological examinations of the spleen under the light microscope revealed degenerative changes. Spleens appeared atrophic in the 200 mg/kg and 400 mg/kg ATR groups, characterized by the effacement of germinal centers, diminution of white pulp, and congestion of red pulp (Figure 1). No significant changes were observed in the control and 100 mg/kg ATR groups. Transmission electron microscopy showed wide karyopyknosis, perinuclear cistern widening, mitochondrial degeneration, and formation of apoptotic bodies in the lymphocytes, all of which were characteristics of cells undergoing apoptosis. These changes were notable in the spleen with dosages of 200 mg/kg and 400 mg/kg ATR groups (Figure 2).

### Flow cytometry

**Figure 3:**
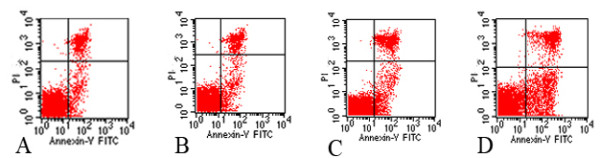
**Flow cytometry plots of splenocytes apoptosis**. The splenocytes were isolated from mice treated with atrazine (2-chloro-4-ethytlamino-6-isopropylamine-1,3,5-triazine; ATR) for 21 days. The annexin-V and propidium iodide (PI) fluorescence was measured using flow cytometer. Results were expressed as dot plots. **(a) **Control group; **(b) **100 mg/kg ATR; **(c) **200 mg/kg ATR; **(d) **400 mg/kg ATR.

**Figure 4:**
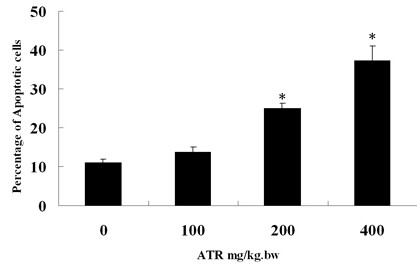
**Histogram showing the percentage of splenocytes exhibiting apoptosis induced by atrazine (2-chloro-4-ethytlamino-6-isopropylamine-1,3,5-triazine; ATR) in mice (n = 10)**. With the increasing concentration of ATR, the apoptotic percentage of splenocytes increased. *P <0.05, compared to control group.

Analysis by flow cytometry revealed that the percentage of apoptotic lymphocytes in the spleen was significantly increased in 200 mg/kg and 400 mg/kg ATR treatment groups, which there were statistic difference with the comparison to the control group (*P *<0.05). In the 400 mg/kg ATR-treated group, the percentage of apoptotic lymphocytes increased fourfold compared to the control group (Figures 3 and 4).

### Expression of apoptotic proteins

**Figure 5:**
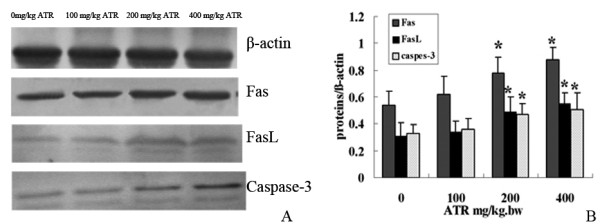
**Western blot gel (a) and histogram (b) showing relative expression of Fas, FasL and active caspase-3 proteins in the spleen**. (a) Western blot showed that the expression of Fas, FasL and active caspase-3 were upregulated with the increasing concentrations of atrazine (2-chloro-4-ethytlamino-6-isopropylamine-1,3,5-triazine; ATR) (0, 100, 200 and 400 mg/kg). β Actin was used as the internal control. (b) Quantification analysis of the western blot. The results were expressed as the ratio of target protein/β actin in each group. *P <0.05, compared to control group.

The western blot showed a dose-dependent increased expression of Fas, FasL and active caspase-3 proteins in 200 mg/kg and 400 mg/kg ATR-treated groups, compared to control group (Figure 5). The β actin control remained constant as expected.

## Discussion

The immune system is a complex network of interacting regulatory genes, hormones, and cells that has evolved in multicellular organisms for the purpose of maintaining a homeostasis against a dynamic battery of foreign environmental agents and/or pathogens. Environmental pollutants can interfere with the normal operation of the immune system leading to a broad range of disorders, including endocrine dysfunction, tumorigenesis, increased rates of inflammatory infectious, and autoimmune diseases. Widespread use of the herbicide ATR increases the chance and rates of exposure to another foreign agent that immune system must contend with. Immunotoxicity of ATR manifests as a decrease in immune response capacity, including the suppression of immune cell function, atrophy of immune organs [24-26]. This study adds to a growing list of publications reporting on the adverse physiological effects of ATR. Surprisingly, overt changes in outward appearance or behavior were not observed in mice administered even the highest ATR dosage. However, spleen and thymus weights decreased significantly in a dose-dependent manner, apoptosis of lymphocytes was recorded in the spleen, and an altered apoptotic protein pathway occurred in mice administered a range of ATR dosages.

Experimental dosage amounts given to mice ranged from 100 mg/kg to 400 mg/kg. These amounts are based on the concentrations used in previous studies [26,27] and are higher than might be expected in natural conditions. However, these amounts ensured the desired effect for the purposes of this study. In California, the potential occupational exposure to atrazine was assessed during mixing, loading and application to field corn and it was estimated that absorbed daily dosages (ADD) of ATR for a mixer-loader-tender applicator was 1.8-6.1 μg/kg/day for short-term exposure (15-21 days) [36,37]. The ADD of ATR is expected to be higher in commercial applicators and farmers in developing country due to inappropriate personal protective equipment and unintentional overspray. Atrazine is not removed from the body within 24 h and its metabolites can still be detected in the urine 48 h after a dose to humans [38]. It is therefore possible that some kinds of effect could occur after repeated dosing and result from an accumulation of chemical above a critical threshold.

Abnormal weight of the spleen and thymus may be an important indicator and a simple method to investigate potential cases of ATR immunotoxicity. The decrease of immune organ weight may be associated with the inhibition of lymphocytes proliferation and/or the increase of lymphocyte death in the spleen and thymus [39,40]. Closer examination using microscopy revealed degenerative histopathological alterations, although this study does not report the functional immunity of the spleen directly. These results do provide, however, indirect evidence that ATR exposure has an adverse impact on the functional immunity of the spleen in mice. This conclusion is supported by other studies reporting on the adverse effects of ATR on the immune system [26,27].

The role of toxin-induced apoptosis has been a major theme in toxicological evaluation, although the relative contribution of apoptosis caused by toxins remains unclear. Studies have shown a cytotoxic effect of ATR on aquatic organisms and amphibians mediated by inducing apoptosis [33,41]. This is the first study that we are aware that identifies ATR-induced apoptosis in a rodent. Splenocytes exhibited the morphological phenotype of apoptosis after ATR exposure and the percentage of apoptotic lymphocytes increased in a dose-dependent manner. Cellular scale ATR-induced apoptosis provides a reasonable explanation for the noted decrease in spleen weight and atrophy in the germinal center of the spleen.

The main aim of this study was to test for possible immunotoxic mechanisms operating at a subcellular scale using a western blot analysis focusing on proteins (Fas, FasL, and caspase-3) that have a central role in the execution of cellular apoptosis. Our results show ATR exposure increases Fas and FasL protein expression in the spleen in dose-dependent manner, which suggests the following immunotoxic mechanisms take effect. The spleen is a peripheral lymphoid organ where lymphocytes are activated upon being presented with foreign antigens. Activated lymphocytes are sensitive to signals that lead to apoptosis [42]. When the peripheral lymphocytes are activated following stimulation with a foreign antigen, such as ATR, lymphocyte apoptosis becomes activated. The fine balance between antiapoptotic and apoptotic genes and/or proteins is altered, which leads to the activation of caspases through cascade reaction. Among caspases, active caspase-3 is the key executioner of apoptosis because it is the final effecter enzyme (and also one of the most important) for shearing chromatins [43]. Our results from western blotting shows that there is a higher expression of active caspase-3 in the spleen of mice after ATR exposure. Consequently, lymphocytes exhibit the morphological characteristics of cells undergoing apoptosis, such as karyopyknosis, apoptotic bodies, and so on. Hence, the immune function of the spleen is impaired by the death of lymphocytes.

## Conclusions

This study provides the first evidence of ATR inducing apoptosis of lymphocytes in a rodent. This indicates the existence of a novel cytotoxic mechanism caused by ATR. Mice administered ATR dosages experience upregulation of Fas/FasL and active caspase-3 proteins. This suggests an immunotoxicological linkage to one of the key lymphocyte immunity regulatory systems. However, many molecular mechanisms involved in this process still remain unclear. Therefore, further research is needed to elucidate more fully the mechanisms of the deleterious effects of ATR on the immune system and to investigate prolonged exposure under natural field conditions.

## Competing interests

The authors declare that they have no competing interests.

## Authors' contributions

XZ participated in the experimental design, preparation of the manuscript, and performed the apoptosis assays. MW was responsible for animal care and executed the western blot. SG assisted in the preparation of splenocytes and apoptosis determination. RR and JZ prepared the histopathological examinations and executed the statistical analysis. This study and its design were conceived by YZ, who coordinated the project, participated in the experimental design, and helped to draft the manuscript. All authors have read and approved the final manuscript.

## Author information

XZ, SG, RR and YZ are from the College of Public Health, Harbin Medical University. Their research area focuses on environmental toxicology. MW and JZ's research interests, respectively, include toxicology and occupational health, and they work for the Centre of Disease Control of Heilongjiang Province in China.

## Pre-publication history

The pre-publication history for this paper can be accessed here:

http://www.biomedcentral.com/1741-7015/9/117/prepub
